# Prevalence and correlates of HIV-risky sexual behaviors among students attending the Medical and Social Welfare Center of the University of Maroua, Cameroon

**DOI:** 10.1186/s13104-015-1638-2

**Published:** 2015-11-02

**Authors:** Jean Jacques N. Noubiap, Jobert Richie N. Nansseu, Shalom Tchokfe Ndoula, Binhuan Wang, Ahmadou M. Jingi, Jean Joel R. Bigna, Leopold N. Aminde, Rosette Amélie Youmbi, Joël Fokom-Domgue

**Affiliations:** Department of Medicine, Groote Schuur Hospital and University of Cape Town, 7925 Observatory, Cape Town, South Africa; Medical Diagnostic Center, Yaoundé, Cameroon; Intensive Care Unit, Mother and Child Centre, Chantal Biya Foundation, Yaoundé, Cameroon; Guidiguis Health District, Guidiguis, Cameroon; Medical and Social Welfare Center of the University of Maroua, Maroua, Cameroon; Department of Population Health, Division of Biostatistics, New York School of Medicine, New York, USA; Department of Internal Medicine and Specialties, Faculty of Medicine and Biomedical Sciences, University of Yaoundé I, Yaoundé, Cameroon; Department of Epidemiology and Public Health, Pasteur Center of Cameroon, PO Box 1274, Yaounde, Cameroon; Clinical Research Education, Networking & Consultancy (CRENC), Douala, Cameroon; Department of Obstetrics and Gynecology, Faculty of Medicine and Biomedical Sciences, University of Yaoundé I, Yaoundé, Cameroon

**Keywords:** Sexual behavior, University students, Cameroon, Africa

## Abstract

**Background:**

Data on sexual behaviors in Cameroonian youths are needed to design and implement effective preventive strategies against HIV/AIDS. This study aimed at assessing sociodemographic and religious factors associated with sexual behaviors among university students in Cameroon.

**Methods:**

In 2011, 411 university students were surveyed by a self-administered questionnaire at the Medical and Social Welfare Center of the University of Maroua. Logistic regression analyses were used to determine correlates of sexual behaviors.

**Results:**

80.8 % of students were sexually active. The mean age at sexual debut was 18.1 years (SD = 3.1). The frequency of premarital sex was 92.8 %. Pornography viewing [adjusted odds ratio (aOR): 4.0, 95 % CI 2.1–7.6; *p* < 0.0001] and an increased age of 1 year (aOR: 1.3, 95 % CI 2.0–7.6; *p* < 0.0001) were significantly associated with having previously had sex. The likelihood to have a lower (<18) age at sexual debut was increased by male gender (aOR: 2.5, 95 % CI 1.7–5; *p* < 0.001), and urban origin (aOR: 2.9, 95 % CI 1.5–5.7; *p* < 0.01). The probability to have a high number (#3) of lifetime sexual partners was increased by age (aOR: 1.1, 95 % CI 1.0–1.2; *p* < 0.001), pornography viewing (aOR: 4.3, 95 % CI 1.9–9.5; *p* < 0.001), an early sexual debut (aOR: 2.8, 95 % CI 1.6–5.0; *p* < 0.001), having had occasional sexual partners (aOR: 7.0, 95 % CI 3.7–13.1; *p* < 0.0001), and was decreased by Muslim religious affiliation (aOR: 0.2, 95 % CI 0.1–0.9; *p* < 0.05). Having had casual sexual partners was associated with less inconsistent condom use (aOR: 0.5, 95 % CI 0.2–0.9; p < 0.05).

**Conclusions:**

Our findings indicate that there is an alarming level of risky sexual behaviors among the study population. Strong and efficient measures should be undertaken to handle such harmful behaviors, this for the prevention and control of HIV/AIDS and other STIs in this vulnerable population.

## Background

Important goals have been achieved in the fight against HIV/AIDS pandemic especially during this last decade. The number of people dying from AIDS-related morbidities worldwide fell to 1.6 million in 2012, down from a peak of 2.3 million in 2005 [1]. Sub-Saharan Africa (SSA) remains disproportionately affected by HIV. In 2012, about 68 % of all people living with HIV and 70 % of those newly infected resided in SSA, a region with only 12 % of the global population. In Cameroon, the prevalence of HIV in the general population has decreased from 5.2 % in 2001 to 4.5 % in 2012, but despite a 27.4 % decline of the total number of new infections during the same period, the infection remains a major public health concern in the country [1].

Nearly half of new HIV infections occur in young people aged 15–24 years [1]. This group represents the most sexually active part of the population and is very sensitive to changes in sexual behaviors [1]. Changing HIV-risky sexual behaviors in the young population is therefore crucial in declining the pandemic. Behavioral interventions to reduce risky sexual behaviors and avert sexually transmitted infections (STIs) and HIV have been primarily based on the so-called ABC strategy: Abstinence from sex before marriage, Being faithful to one’s partner, and using Condoms [2]. These socio-behavioral interventions alongside other prevention efforts have borne fruit, with the declining rates of new HIV infections among youths being the testimony of these interventions [1, 2].

Since the advent of HIV/AIDS, the number of studies in this field has grown, with focus on identifying, explaining, and changing sexual practices relevant to HIV transmission. Some studies have shown the impact of financial and education levels of the household, and the influence of urban or rural settings upon sexual behaviors [3, 4]. Other factors that have been shown to influence sexual behaviors include living arrangements, alcohol consumption, substance abuse, lack of parental control, peer pressure [4–9]. Religion was found to be a protective factor in sexual abstinence among young people in Zimbabwe and Cote d’Ivoire [8, 10]. By contrast, an investigation of university students in Nigeria did not show any association between religion and sexual behavior [7]. The importance of these factors on HIV-risky sexual behaviors varies thereby between settings [3–11].

In Cameroon, the mean HIV prevalence is 4.5 %, with respectively 1.8 and 1.0 % of young females and males aged 15–24 being infected [1]. The youths aged 15–24 years represent an important part of the national population, and the majority of university students belong to this age group. University students are often regarded as being at a higher risk of acquiring HIV infection, hence they are categorized under the most at risk population segments (MARPS) owing to their inclination to be engaged in risky sexual behaviors and to their sense of non-vulnerability [12]. Consequently, university students represent a target population of national HIV prevention programs in order to effectively reduce the incidence of HIV infection nationwide. Behavioral factors relevant to HIV risks among university students need to be identified and clarified in order to develop evidence-based prevention strategies against HIV/AIDS and other STIs in this population. To the best of our knowledge, there is no published study that has investigated the prevalence and correlates of sexual behaviors among university students in Cameroon. This study therefore aimed to assess sociodemographic and religious factors associated with sexual behaviors among university students in Cameroon.

## Methods

### Ethical considerations

The study was granted ethical approval by the Institutional Review Board of the Medical and Social Welfare Center of the University of Maroua, Cameroon. This ethical approval included the use of data from university students younger than 18. The study was performed in accordance with the guidelines of the Helsinki Declaration. Written informed consent was obtained from all the participants.

### Study population and setting

We conducted a cross-sectional study in October 2011 at the Medical and Social Welfare Center of the University of Maroua. This is a public university situated in Maroua, headquarter of the Far-North Region of Cameroon, which has a population estimated at 200,000 inhabitants [13]. Founded in 2008, the University of Maroua receives students from all over the country with various sociocultural backgrounds that may impact the local behaviors, hence the epidemiology of HIV in the city. At the time the study was conducted, there were 14,556 students in this university. The mean student age was 23.9 (SD 5.3) years, with 67.1 % (n = 9767) of males and 69.7 % (10,145) of undergraduate students.

### Data collection

Data were collected using a structured pretested self-administered questionnaire consisting of 49 questions. This instrument was prepared based on validated questionnaires used in some previous studies [3, 14, 15], and considered by a panel of consulting experts. To assess face and content validity, the questionnaire was pre-tested by 25 students (15 undergraduates and 10 graduates in different field of studies) for readability, relevance, language, comprehension, and cultural appropriateness. These students were requested to take note of any item of the questionnaire that they found difficult to understand. Problematic items were detected during group discussions, and the questionnaire was revised accordingly.

This questionnaire assessed sociodemographic and religious factors as well as sexual behaviors. During the study period, all the university students who visited the Medical and Social Welfare Center of the University of Maroua for a systematic and free of charge medical evaluation, counting for their obligatory yearly medical check-up, were proposed to participate in the study, and consenting students were consecutively enrolled. Prior to the distribution of the study questionnaire, students were orally informed about the purpose of the study and were given instructions to fill them in after we have obtained a written and signed informed consent from each of them. The questionnaires were anonymous. We excluded from the analysis questionnaires that were incompletely filled (i.e. less than 90 % of questions answered).

### Definition of variables

Most of the variables were selected and defined in accordance with the study by Agardh et al. [3].

#### Background variables

Area of origin was dichotomized into ‘‘rural’’ and ‘‘urban’’. The educational level of the head of household during childhood was dichotomized, so that ‘‘did not finish primary school’’ and ‘‘completed primary school’’ were coded as ‘‘low’’, and ‘‘completed secondary school’’ and any education above that was classified as ‘‘high’’. The marital status of parents of household during childhood was defined as “monogamous”, “polygamous” and “single parent”. The accommodation of the participant at the moment of the study was reported as “single” if he/she was living alone, “in collocation” if he/she was sharing accommodation with one or several other students and “family” if he/she was living in his/her family. The religion of the participant was reported by selecting one of the following alternatives: ‘‘Catholic’’, ‘‘Protestant’’, ‘‘Muslim’’, ‘‘Pentecostist’’, or ‘‘others’’. The marital status of the participant was dichotomized as ‘‘married’’ or ‘‘single’’. Faculty of study was reported and categorized as ‘‘Science’’ or ‘‘Humanities, arts and social sciences’’. The current academic level was categorized as ‘‘undergraduate’’ for level ≤3 or “graduate” for level ≥4.

#### Sexual behavior variables

The variable for having previously had sexual intercourse was defined as ‘‘yes’’ or ‘‘no’’, based on responses to the question: ‘‘have you ever had sexual intercourse?’’. Age at sexual debut was dichotomized, so that having sexual intercourse for the first time before age 18 was coded as ‘‘low’’, while having sexual intercourse for the first time at or above age 18 was coded as ‘‘high’’. The variable “lifetime sexual partners” referred to the total number of partners the student has ever had, and was defined by responses to the question: ‘‘how many sexual partners have you ever had?’’ This variable was then dichotomized, so that ≥3 was coded as ‘‘high’’, and <3 was coded as ‘‘low’’. Inconsistent condom use with new partner was dichotomized as ‘‘yes’’ or ‘‘no’’. The variables for having had sexual education at school, for having previously discussed with parents about sexual issues, for having previously viewed pornography, for having had occasional sexual partner, for having used condom and for having drunk alcohol before the first sexual intercourse were defined as ‘‘yes’’ or ‘‘no’’. Age at first pornographic viewing was dichotomized, so that pornography viewing for the first time before age 18 was coded as ‘‘low’’, and at or above age 18 as ‘‘high’’. Pornography viewing was defined as viewing printed or visual material containing the explicit description or display of sexual organs or activity, intended to stimulate sexual excitement.

### Data analysis

Data were coded, entered and analyzed using the Statistical Analysis System (SAS) version 9.3 (SAS Institute Inc, Cary, NC, USA). We described categorical variables using their frequencies and percentages. The Chi-square test and the *t* test were used to compare male and female with respect to the prevalence of studied variables. Odds ratios (OR) with 95 % confidence interval (CI) were used to evaluate the factors associated with having previously had sex, low age at sexual debut, high number of lifetime sexual partners, and inconsistent of condom use. Unadjusted ORs were calculated using logistic regressions. Furthermore, we considered a variable selection issue due to the large number of available factors. By firstly postulating four candidate models according to existing literature and common sense, and a fifth model including variables with a p < 0.2 in univariate analyses; a series of logistic regression analyses were performed, which were compared according to the Akaike information criterion (AIC). The optimal model among the candidates is the one with the smallest AIC score [16]. We also used a pre-defined p-value of 0.2 as a rule of thumb to select variables to be included in the model for adjustments. This manuscript was written following STROBE guidelines for the reporting of observational studies [17].

## Results

### Background characteristics

During the study period, we approached 452 students of whom 31 refused to take part in the study, hence a response rate of 93.1 %. Besides, 10 questionnaires were incompletely filled and were consequently rejected. On the whole, 411 students, who responded to 90 % or more of the questions, were included in the study. Their ages ranged from 17–50 years, with a mean of 24.6 (SD 4.6) and a median of 24 (interquartile range 22–26). Two hundred and seventy-one students (65.9 %) were male. Most of the respondents were coming from an urban area (83.9 %), were undergraduate (68.4 %), single (88.3 %), and living outside the family (80.8 %). The majority of students (72.5 %) grew up in a family with a head of household who had a high educational level (secondary school or above). Catholicism and Protestantism were the most frequent religious affiliations, representing respectively 46.9 and 32.6 % of the study population. Other socio-demographic characteristics are presented in Table 1.

**Table 1:** Socio-demographic characteristics and sexual behavior factors

Variables	All (n = 411)	Male (n = 271)	Female (n = 140)	*p* value
	N (%) or mean (standard deviation)^a^			
Sex				
Male	271 (65.9)			
Female	140 (34.1)			
Age (years)	24.6 (4.6)	25.2 (5.0)	23.5 (3.4)	0.201
Area of origin				
Urban	345 (83.9)	217 (80.1)	128 (91.4)	0.004
Rural	66 (16.1)	54 (19.9)	12 (8.6)	
Marital status of parents of household				
Monogamous	241 (58.6)	144 (53.1)	97 (69.3)	0.002
Polygamous	135 (32.8)	105 (38.8)	30 (21.4)	
Single parent	35 (8.5)	22 (8.1)	13 (9.3)	
Educational level of head of household				
Low (less than primary)	113 (27.5)	87 (32.1)	26 (18.6)	0.004
High (secondary and higher)	298 (72.5)	184 (67.9)	114 (81.4)	
Academic level				
Undergraduate	281 (68.4)	196 (72.3)	85 (60.7)	0.017
Graduate	130 (31.6)	75 (27.7)	55 (39.3)	
Type of studies				
Science	195 (47.4)	140 (51.7)	55 (39.3)	0.017
Humanities, arts and social sciences	216 (52.6)	131 (48.3)	85 (60.73)	
Accommodation				
Single	189 (46.0)	120 (44.3)	69 (49.3)	0.002
Collocation	143 (34.8)	109 (40.2)	34 (24.3)	
In family	79 (19.2)	42 (15.5)	37 (26.4)	
Marital status				
Married	48 (11.7)	31 (11.4)	17 (12.1)	0.833
Single	363 (88.3)	240 (88.6)	123 (87.9)	
Religious affiliation				
Catholic	193 (46.9)	121 (44.7)	72 (51.5)	0.410
Protestant	134 (32.6)	91 (33.6)	43 (30.7)	
Muslim	48 (11.7)	31 (11.4)	17 (12.1)	
Pentecostal	36 (8.8)	28 (10.3)	8 (5.7)	
Had sexual education at school				
Yes	289 (70.3)	184 (67.9)	105 (75.0)	0.136
No	122 (29.7)	87 (32.1)	35 (25.0)	
Had ever discussed with parent about sexuality				
Yes	228 (55.5)	137 (50.6)	91 (65.0)	0.005
No	183 (44.5)	134 (49.4)	49 (35.0)	
Had ever watched pornography				
Yes	330 (80.3)	236 (87.1)	94 (67.1)	<0.0001
No	81 (19.7)	35 (12.9)	46 (32.9)	
Age at the first pornographic viewing				
Low (<18)	195 (59.1)	146 (61.9)	49 (52.1)	0.105
High (≥18)	135 (40.9)	90 (38.1)	45 (47.9)	
Previously had sex				
Yes	332 (80.8)	225 (83)	107 (76.4)	0.109
No	79 (19.2)	46 (17)	33 (23.6)	
Age at sexual debut				
Low (<18)	184 (55.4)	137 (60.9)	47 (43.9)	0.004
High (≥18)	148 (44.6)	88 (39.1)	60 (56.1)	
Condom use during the first sexual intercourse				
Yes	188 (56.6)	109 (48.4)	79 (73.8)	<0.0001
No	144 (43.4)	116 (51.6)	28 (26.2)	
Had drunk alcohol before the first sexual intercourse				
Yes	21 (6.3)	18 (8)	3 (2.8)	0.072
No	311 (93.7)	207 (92)	104 (97.2)	
Sexual partner of the first sexual intercourse				
Spouse	24 (7.2)	11 (4.9)	13 (12.1)	<0.0001
Boy/girlfriend	237 (71.4)	148 (65.8)	89 (83.2)	
Prostitute	10 (3.0)	10 (4.4)	0 (0)	
Others	61 (18.4)	56 (24.9)	5 (4.7)	
Number of lifetime sexual partner				
Low (<3)	121 (36.4)	64 (28.4)	57 (53.3)	<0.0001
High (≥3)	211 (63.6)	161 (71.6)	50 (46.7)	
Having had casual sexual partner				
Yes	163 (49.1)	142 (63.1)	21 (19.6)	<0.0001
No	169 (50.9)	83 (36.9)	86 (80.4)	
Inconsistent condom use with new sexual partner				
Yes	144 (43.4)	116 (51.6)	28 (26.2)	0.302
No	188 (56.6)	109 (48.4)	79 (73.8)	

^a^Categorical variables are described using their frequencies and percentages (in brackets); continuous variables are described using their means and standard deviations

### Sexual behaviors

Most of the students have had sexual education at school (70.3 %), but only 55.5 % had ever discussed with their parents about sexuality, with significantly more females than males (*p* = 0.005). Pornography viewing was frequent (80.3 %) with a significant male predominance (*p* < 0.0001). A dominating number of students (80.8 %) reported having previously had sexual intercourses, 55 % of them before age 18. The mean age at sexual debut was 18.1 years (SD = 3.1). More men had early sexual debut (*p* = 0.004). The frequency of premarital sex was 92.8 % with a male predominance (*p* < 0.0001). Having not used condom during the first sexual intercourse was frequent (43.4 %), especially in males (*p* < 0.0001). The number of lifetime sexual partners was greater in males (*p* < 0.0001), with 63.6 % of students having had 3 or more lifetime sexual partners. Slightly less than half of students reported having had at least one occasional sexual partner, mostly males (*p* < 0.0001). Inconsistent condom use was reported by 27.1 % of students with no gender difference (*p* = 0.302) (Table 1).

Unadjusted correlates of having previously had sex included age (*p* < 0.0001), being married (*p* < 0.0001), single and collocation accommodation (*p* < 0.05), protestant affiliation (*p* < 0.05) and pornography viewing (*p* < 0.0001) (Table 2). In logistic regression analyses adjusted for multiple variables, we compared five candidate models using AIC (the smaller the better) (Table 3). It seems that model 3 is the best model among the five candidates. Inference based on this model showed that students who have viewed pornography were more likely to have previously had sex than those who have not viewed pornography [adjusted odds ratio (aOR): 3.4, 95 % CI 1.8–6.4; *p* < 0.0001], and each increment of 1 year of age was associated with a 1.3 times increased likelihood of having previously had sex (95 % CI 1.2–1.4; *p* < 0.0001).

**Table 2:** Unadjusted correlates of having previously had sex, low age at sexual debut, high number of lifetime sexual partners and inconsistent condom use

Explanatory variables (referent)	Dependent variables OR (95 % CI)			
	Having previously had sex (n = 411)	Low age at sexual debut (n = 332)	High number of lifetime sexual partners (n = 332)	Inconsistent condom use (n = 332)
Age (increase by 1 year)	1.3 (1.1–1.4)****	1.0 (0.9–1.1)	1.1 (1.0–1.1)****	1.0 (0.9–1.1)
Female (male)	0.7 (0.4–1.2)	0.5 (0.3–0.8)**	0.3 (0.2–0.5)****	1.0 (0.6–1.6)
Collocation accommodation (in family)	1.9 (1.0–3.8)*	1.8 (0.9–3.4)	2.2 (1.1–4.3)*	0.7 (0.3–1.3)
Single accommodation (in family)	2.0 (1.0–3.7)*	1.9 (1.0–3.6)*	1.8 (1.0–3.4)	0.8 (0.4–1.6)
Unmarried (married)	0.001 (0.001–999)***	1.2 (0.6–2.3)	0.5 (0.3–1.0)*	0.2 (0.1–0.4)****
Urban (rural)	1.1 (0.6–2.1)	2.5 (1.4–4.6)**	1.1 (0.6–2.1)	0.5 (0.3–1.0)*
Polygamous (monogamous)^a^	1.0 (0.6–1.7)	1.2 (0.7–1.9)	1.1 (0.7–1.7)	0.8 (0.5–2.7)
Single parent (monogamous)^a^	2.6 (0.8–8.8)	1.0 (0.5–2.2)	1.7 (0.7–4.1)	0.9 (0.4–2.1)
Low educational level of head of household (high)	0.7 (0.4–1.2)	0.5 (0.3–0.9)*	0.8 (0.5–1.5)	1.3 (0.8–2.3)
Academic level (undergraduate)	0.9 (0.5–1.5)	0.8 (0.5–1.3)	0.9 (0.6–1.5)	1.0 (0.6–1.7)
Types of studies (sciences)	1.0 (0.6–1.1)	0.8 (−0.5 to 1.3)	1.0 (0.6–1.5)	1.1 (0.7–1.8)
Catholic affiliation (Pentecostal)	1.5 (0.6–3.3)	0.6 (0.3–1.5)	0.4 (0.1–1.1)	0.7 (0.3–1.7)
Muslim affiliation (Pentecostal)	1.7 (0.6–4.7)	1.1 (0.4–3.0)	0.2 (0.1–0.7)**	1.0 (0.3–2.9)
Protestant affiliation (Pentecostal)	2.5 (1.0–5.9)*	0.6 (0.2–1.4)	0.5 (0.2-1.4)	1.0 (0.4–2.5)
Had sexual education at school (yes)	1.1 (0.7–2.0)	1.2 (0.8–2.0)	1.1 (0.7–1.8)	1.4 (0.8–2.3)
Had ever discussed with parent about sexuality (yes)	1.0 (0.6–1.6)	0.8 (0.5–1.2)	0.7 (0.5–1.2)	1.9 (1.1–3.0)*
Had ever watched pornography (no)	3.2 (1.9–5.6)****	1.2 (0.7–2.2)	4.7 (2.5–8.8)****	0.5 (0.2–0.8)**
Age at sexual debut (high)			2.9 (1.8–4.7)****	0.9 (0.6–1.5)
Previously had casual sexual partners (no)			10.5 (6.0–18.4)****	0.5 (0.3–0.8)**
Number of lifetime sexual partners (low)				0.6 (0.4–1.0)*
Inconsistent condom use (no)			0.6 (0.4–1.0)*	

*OR* unadjusted odds ratio

^a^ Marital status of parents of household during childhood

* *p* < 0.05; ** *p* < 0.01; *** *p* < 0.001; ^****^ *p* < 0.0001

**Table 3:** Adjusted correlates of having previously had sex, low age at sexual debut, high number of lifetime sexual partners and inconsistent condom use

Explanatory variables (referent)	Dependent variables aOR (95 % CI)			
	Having previously had sex (n = 411)	Low age at sexual debut (n = 332)	High number of lifetime sexual partners (n = 332)	Inconsistent condom use (n = 332)
Age (increase by 1 year)	1.3 (1.2–1.4)****	1.0 (0.9–1.0)	1.1 (1.0–1.2)^***^	1.0 (0.9–1.1)
Female (male)	1.3 (0.7–2.5)	0.4 (0.2–0.6)***	1.0 (0.6–1.9)	
Collocation accommodation (in family)	1.5 (0.7–3.2)	1.6 (0.8–3.2)		
Single accommodation (in family)	1.8 (1.8–3.6)*	1.7 (0.9–3.3)		
Unmarried (married)				0.3 (0.2–0.8)*
Urban (rural)		2.9 (1.5–5.7)**		0.5 (0.3–1.0)
Polygamous (monogamous)^a^				
Single parent (monogamous)^a^				
Low educational level of head of household (high)		0.6 (0.3–1.0)		
Academic level (undergraduate)				
Types of studies (sciences)				
Catholic affiliation (Pentecostal)			0.7 (0.2–2.2)	
Muslim affiliation (Pentecostal)			0.2 (0.1–0.9)*	
Protestant affiliation (Pentecostal)			0.8 (0.2–2.8)	
Had sexual education at school (yes)		1.4 (0.9–2.5)		1.1 (0.6–1.9)
Had ever discussed with parent about sexuality (yes)		0.8 (0.5–1.3)		1.5 (0.9–2.6)
Had ever watched pornography (no)	3.4 (1.8–6.4)****		4.3 (1.9–9.5)***	0.7 (0.3–1.3)
Age at sexual debut (high)			2.8 (1.6–5.0)***	0.9 (0.6–1.5)
Previously had casual sexual partners (no)			7.0 (3.7–13.1)****	0.5 (0.3–0.9)*
Number of lifetime sexual partners (low)				0.6 (0.4–1.0)*
Inconsistent condom use (no)			0.7 (0.4–1.3)	

The best model selected based on the Akaike Information Criterion is presented for each outcome variable

*aOR* adjusted odds ratio

^a^ Marital status of parents of household during childhood

* *p* < 0.05; ** *p* < 0.01; *** *p* < 0.001; ^****^ *p* < 0.0001

Being female (*p* < 0.01), urban origin (*p* < 0.01), single accommodation (*p* < 0.05) and the higher level of education of the head of household during childhood (*p* < 0.05) were found to be significantly associated with a low age at sexual debut in univariate analysis (Table 2). In the adjusted analyses, the best candidate model (model 5) showed that students from an urban area (aOR: 2.9, 95 % CI 1.5–5.7; *p* < 0.01) were more likely to have had their first sexual intercourse before age 18, while females were less likely to have low age at first sexual intercourse (aOR: 0.4, 95 % CI 0.2–0.6; *p* < 0.001) (Table 3).

As depicted by Table 2, in the unadjusted analyses a high number of lifetime sexual partners was associated with age (*p* < 0.0001), male sex (*p* < 0.0001), collocation accommodation (*p* < 0.05), being married (*p* < 0.05), Muslim religious affiliation (*p* < 0.01), pornography viewing (*p* < 0.0001), age at sexual debut (*p* < 0.0001), having had occasional sexual partners (*p* < 0.0001), and inconsistent condom use (*p* < 0.05). In the adjusted analyses, the likelihood to have a high number of lifetime sexual partners was increased by age (aOR: 1.1, 95 % CI 1.0–1.2; *p* < 0.001), pornography viewing (aOR: 4.3, 95 % CI 1.9–9.5; *p* < 0.001), an early sexual debut (aOR: 2.8, 95 % CI 1.6–5.0; *p* < 0.001) and having had occasional sexual partners (aOR: 7, 95 % CI 3.7–13.1; *p* < 0.0001). Compared to students of Pentecostal faith, Muslims (aOR: 0.2, 95 % CI 0.1–0.9; *p* < 0.05) were significantly less likely to have a high number of lifetime sexual partners (Table 3).

Unadjusted correlates of inconsistent condom use were being married (*p* < 0.0001), rural origin (*p* < 0.05), never having discussed sexuality with parents (*p* < 0.05), pornography viewing (*p* < 0.01), having had occasional sexual partners (*p* < 0.01), and low number of lifetime sexual partners (*p* < 0.05). Adjusted analyses showed that being married (aOR: 0.3, 95 % CI 0.2–0.3; *p* < 0.05) and having had casual sexual partners (aOR: 0.5, 95 % CI 0.3–0.9; *p* < 0.05) decreased the likelihood of inconsistent condom use (Table 3).

## Discussion

Data on sexual behaviors in youths are important to develop evidence-based prevention strategies against HIV/AIDS and other sexually transmitted infections in this population. Such data are scarce in the Cameroonian population. A recent study among 1st year Cameroonian university students appraised their knowledge, attitudes and practice pertaining to condom perception and prevention of HIV/AIDS [18]. However, the present study was the first to investigate the prevalence and correlates of sexual behavior factors in university students in Cameroon.

We found that 80.8 % of students have had sexual intercourse. A significantly lower rate has been found in a nationwide survey in China where only 11.3 % had experienced sexual intercourse [19]. This low rate in China may be explained by the Confucian-based traditional culture expecting in terms of sexuality, that men and women should conduct themselves properly from an emotional distance at all times, hence avoiding any contact before marriage [20]. For example, the Chinese Ministry of Education prohibited marriage among university students until 2005 and the universities offered a context discouraging university students’ sexual activities. In China, many universities have direct and indirect regulations that limit intimate relationships between students of opposite sex in school [15]. Rates lower than ours have also been found in other African countries. In Uganda for instance, 59 % of university students reported having previously had sexual intercourse, only 28 % of undergraduate students in a study in Ethiopia [3, 14]. Differences in the rate of sexual intercourse between the aforementioned studies and ours could be explained by the lower mean age of participants in these studies. Indeed, age appeared to have an important influence on sexual debut in our study, as a 1 year increase in age was associated with 30 % increased odds of having previously had sex.

On the other hand, the rate of sexual experience found in our study is similar to those of Western countries. Indeed, a study in the United States found that 82.1 % of university students had experienced heterosexual intercourse [21]. Likewise, 74 % of university students reported ever having had sexual intercourse in Turkey [22]. These similarities could reflect the influence of sexual-related western culture on Cameroonian youths, through mass media like the internet and television. A high number of television programs in western countries have a sexual content, even slight, and thereby enhance engaging in sexual practices. Although there are some similarities between the western studies and ours, it is worth noting that these studies may have differed in terms of characteristics of the study population, design and setting, and that in Africa, structural determinants such as socio-economic status, gender-power related factors, and socio-cultural factors seem to greatly influence people sexual habits.

Eighty percent of our participants had been exposed to pornography, 59 % of them before 18 years old. In Cameroon, because of the absence of regulations, adolescents can freely buy pornographic videos or magazines at low prices. They can even watch such movies in video clubs without any age restriction, or on the internet. We found that sexual debut was associated with pornography viewing. Furthermore, students who had been exposed to pornography compared to those who had not were significantly more engaged in sexual activities. Much more, pornographic viewing was also associated with a high number of lifetime sexual partners. A recent study has demonstrated among emerging adults in college that more frequent viewing of pornography is associated with a higher incidence of potentially risky sexual behavior (hooking up) and a higher number of unique hook up partners via sexual scripts. The study has also shown that more frequent viewing of pornography is associated with having had more previous sexual partners of all types, more one occasion sexual partners (“one night stands”), and plans to have a higher number of sexual partners in the future [23]. Considering our results, there is an urgent need for a strict regulation on the access to pornographic media in Cameroon.

The mean age at sexual debut in our study, 18.1 years, is in keeping with the 17.5 years found in undergraduate students in Ethiopia as well as the 17.9 years found in university students in Uganda [3, 14]. Mirroring other studies [14, 24], we found that males had a significantly lower age at sexual debut. It has been shown that college students from cities and towns were more prone to sexual intercourses than those originating from a rural milieu [24]. Although area of origin was not associated with the age at sexual debut in this study, students from an urban area had a significantly lower age at sexual debut.

Having had casual sexual partners was frequent (50.9 %), as well as a high number of lifetime sexual partners (63.6 %), with a very strong association between them. As expected, an increment of the participant’s age as well as an early sexual debut increased the likelihood to have a high number of lifetime sexual partners. Religion affiliation was also associated with the number of lifetime sexual partners. Studies in Africa and elsewhere have shown that religious engagement was a protective factor for risky sexual behaviors [25–27]. Religion has been categorized in at least four dimensions: personal devotion (a sense of personal connection to a god), personal conservatism (rigid or literal adherence to the creed of a religious denomination), institutional conservatism (fundamentalism of a religious denomination), and participation in a religious community [28]. A study on religiosity and sexual responsibility among adolescent girls showed that three of these four dimensions (personal devotion, frequent attendance, and institutional conservatism) were linked to a lower number of sexual partners in the previous year [27]. Pentecostal faith has been shown to have a protective effect on the likelihood to have premarital sex and to have a high number of lifetime sexual partners [3, 29, 30]. This could be explained by the fact that Pentecostal churches are very conservative with a strict Christian indoctrination, teaching ‘‘abstinence until marriage’’. Some studies have also shown that Pentecostal youths are less likely to engage in early sexual activities [3, 29, 30]. By contrast, Pentecostal affiliation was not associated to virginity in our study. Moreover, compared to Muslims for instance, students of Pentecostal faith were more likely to have a high number of lifetime sexual partners. These findings could be explained by the fact that probably most of the students of Pentecostal affiliation had joined it after sexual debut, as the growth of Pentecostal churches is quite recent in Cameroon as it was the case with Ugandan students [3].

Inconsistent condom use is a risky sexual behavior. Indeed, consistent use of condoms, irrespective of other risky sexual behaviors, has been demonstrated to be an effective means of preventing the acquisition and transmission of HIV and other STIs [31]. We found that 43.4 % of sexually-experienced students used condom at their first sexual encounter. This is greater than what has been found in similar studies in Ethiopia (32.7 %) and in Kenya (30.1 %) [14, 32]. It is worth noting that inconsistent condom use at first sexual encounter, if considered as a risky behavior, has probably been overestimated in our study because some students probably had their sexual debut in a formal marriage and were less likely to use condom for the reproductive purposes. Furthermore, the rate of inconsistent condom use decreased from 56.6 % at first sexual encounter, to 27.1 % during the following sexual experiences. Perception of the risk of transmission of HIV through unprotected sex may have increased condom use among them. In fact, it has been shown that young men who perceived their risk as high were less likely to report risky behaviors such as inconsistent condom use especially with casual sexual partners [33]. Accordingly, we found that having had casual sexual partners decreased the likelihood of inconsistent condom use. This finding may also be explained by report bias.

Almost two-third of participants reported having had sex education at school, and about half of them had ever discussed sexual issues with their parents. Similarly, a study conducted in Sweden reported that 50 % of high school students had talked with their parents about sex [34]. On the contrary, in China where sex is a taboo in adolescent-parent communication, only 13.7 % of university students discussed with their parents about sex [15]. A large number of studies conducted throughout the world have shown controversies about the influence of sex education on youth’s sexual behaviors. Some of them found that sex education may reduce the level of sexual activities as well as risky sexual behaviors [35, 36]. A randomized controlled trial found that two middle school sexual education programs-a risk avoidance (RA) program and a risk reduction (RR) program-delayed initiation of sexual intercourse (oral, vaginal, or anal sex) and reduced other sexual risk behaviors in students [36].

On the contrary, other studies revealed that sex education might not cause any change in sexual behaviors [37]. Our study showed no association between sex education and sexual behavior such as having previously had sex, age at sexual debut, number of lifetime sexual partners, and consistency of condom use. Although the number of participants who reported having had sexual education at school was relatively high, there was no association between sexual education and any sexual behavior. These findings stress the need for an improvement in the content of the sexual education in our context. Furthermore, sexual and reproductive health services are not delivered in an organized way in Cameroonian universities. Such services would surely be highly beneficial to students.

This study had some limitations. First, its cross-sectional design prevented us from identifying cause-and-effect associations between the variables analyzed. However, most of the explanatory variables were unlikely to be affected by the four main outcome variables. Secondly, we did not investigate homosexual sex. It has been shown for instance that despite the cultural taboo and illegality of homosexuality in African countries, there are some youths practicing it [38]. Thirdly, evaluation of sexual behaviors in this study were based on self-reports. Some participants may have underreported sexual behaviors that would be viewed as socially undesirable. Social desirability scale may be included in future studies. Forth, given our study population was conveniently selected on a consecutive rather than a random basis, it is likely that it did not proportionally represent all the different sub-groups of the entire population of university students in terms of age groups, gender, levels, etc., resulting thereby in selection bias that may have hindered the quality and generalization of our results. Finally, we only included completed surveys in order to assure better accuracy in logistic regression analyses. However, this has led to a response bias. Nonetheless, our study also has major strengths including in particular the use of robust analytical methods to carefully examine the research questions. Furthermore, to the best of our knowledge, this is the first study investigating correlates of risky sexual behaviors among Cameroonian university students.

## Conclusions

Our findings indicate that there is an alarming level of risky sexual behaviors among the study population. Risky sexual behaviors have been found to be significantly associated with pornography viewing. Muslim religion, male gender, urban origin, single accommodation, and higher level of education of the head of household during childhood were other forces associated with sexual behavior among university students in Cameroon. These findings call for an urgent need to undertake robust measures to control pornography viewing among Cameroonian youths. Contextualized messages on sex education should be sent via medias (especially audiovisual medias), with an emphasis on youths living in urban areas. These measures will be of utmost importance for the prevention and control of HIV/AIDS and other STIs in this vulnerable setting.

## References

[1] UNAIDS. UNAIDS report on the global AIDS epidemic 2013. 2013. http://www.unaids.org/en/media/unaids/contentassets/documents/epidemiology/2013/gr2013/UNAIDS_Global_Report_2013_en.pdf. Accessed 31 Oct 2015.

[2] Okware S, Kinsman J, Onyango S, Opio A, Kaggwa P (2005). Revisiting the ABC strategy: HIV prevention in Uganda in the era of antiretroviral therapy. Postgrad Med J.

[3] Agardh A, Tumwine G, Östergren PO (2011). The impact of socio-demographic and religious factors upon sexual behavior among Ugandan university students. PLoS One.

[4] Fentahun N, Mamo A (2014). Risky sexual behaviors and associated factors among male and female students in Jimma zone preparatory schools, south west Ethiopia: comparative study. Ethiop J Health Sci.

[5] Cohen J, Tate T (2006). The less they know, the better: abstinence-only HIV/AIDS programs in Uganda. Reprod Health Matt.

[6] Wellings K, Collumbien M, Slaymaker E, Singh S, Hodges Z (2006). Sexual behaviour in context: a global perspective. Lancet.

[7] Omoteso BA (2006). A study of the sexual behaviour of university undergraduate students in southwestern Nigeria. Soc Sci Med.

[8] Sambisa W, Curtis SL, Stokes CS (2009). ethnic differences in sexual behaviour among unmarried adolescents and young adults in Zimbabwe. J Biosoc Sci.

[9] Sadgrove J (2007). ‘Keeping Up Appearances’: Sex and religion amongst university students in Uganda. J Relig Afr.

[10] Koffi AK, Kawahara K (2008). Sexual abstinence behavior among never-married youths in a generalized HIV epidemic country: evidence from the 2005 Cote d’Ivoire AIDS indicator survey. BMC Public Health.

[11] Letamo G, Mokgatlhe LL (2013). Predictors of risky sexual behaviour among young people in the era of HIV/AIDS: evidence from the 2008 Botswana AIDS Impact Survey III. Afr J Reprod Health.

[12] Taffa N (1998). Sexual activity of out-of-school youth, and their knowledge and attitude about STDs and HIV/AIDS in Southern Ethiopia. Ethiop J Health Dev.

[13] Bureau central des recensements et des études de population du Cameroun. Rapport de présentation des résultats définitifs de 3^ème^ recensement général de la population humaine. http://www.statistics-cameroon.org/downloads/Rapport_de_presentation_3_RGPH.pdf. Accessed 31 Oct 2015.

[14] Dingeta T, Oljira L, Assefa N (2012). Patterns of sexual risk behavior among undergraduate university students in Ethiopia: a cross-sectional study. Pan Afr Med J.

[15] Chi X, Yu L, Winter S (2012). Prevalence and correlates of sexual behaviors among university students: a study in Hefei. China. BMC Public Health.

[16] Bumham KP, Anderson DR: Model selection and multimodel inference: a practical information-theoretic approach. 2002. 2^nd^ ed. New York: Springer Verlag.

[17] von Elm E, Altman DG, Egger M, Pocock SJ, Gøtzsche PC, Vandenbroucke JP (2007). The Strengthening the Reporting of Observational Studies in Epidemiology (STROBE) statement: guidelines for reporting observational studies. Bull World Health Organ.

[18] Sobze Sanou M, Fokam J, Guetiya Wadoum R, Russo G, Onohiol JF (2013). Condom perception and prevention of HIV/AIDS infection in Cameroon: appraisal of knowledge, attitudes and practices among level one students of the University of Dschang. Ig Sanita Pubbl.

[19] Research Group on Sex Education among University Students (2001). Report on sexual behavior survey among Chinese university students in 2000. Youth Study.

[20] Hong W, Yamamoto J, Chang DS (1993). Sex in Confucian society. J Am Acad Psychoanal.

[21] Berg CJ, Lowe K, Stratton E, Goodwin SB, Grimsley L, Rodd J, Williams C, Mattox C, Foster B (2014). Sociodemographic, psychosocial, and health behavior risk factors associated with sexual risk behaviors among southeastern US college students. Open J Prev Med.

[22] Gökengin D, Yamazhan T, Özkaya D, Aytuǧ S, Ertem E, Arda B, Serter D (2003). Sexual knowledge, attitudes, and risk behaviors of students in Turkey. J Sch Health.

[23] Braithwaite SR, Coulson G, Keddington K, Fincham FD (2015). The influence of pornography viewing on sexual scripts and hooking up among emerging adults in college. Arch Sex Behav.

[24] Zuo XY, Lou CH, Gao E, Cheng Y, Niu HF, Zabin LS (2012). Gender differences in adolescent premarital sexual permissiveness in three Asian cities. J Adolesc Health.

[25] Shirazi KK, Morowatisharifabad MA (2009). Religiosity and determinants of safe sex in Iranian non-medical male students. J Relig Health.

[26] McCree DH, Wingood GM, DiClemente R, Davies S, Harrington KF (2003). Religiosity and risky sexual behavior in African-American adolescent females. J Adolesc Health.

[27] Kendler KS, Gardner CO, Prescott CA (1997). Religion, psychopathology, and substance use and abuse; a multimeasure, genetic-epidemiologic study. Am J Psychiatry.

[28] Miller L, Gur M (2002). Religiousness and sexual responsibility in adolescent girls. J Adolesc Health.

[29] Schmalzbauer J (1993). Evangelicals in the new class: class versus subcultural predictors of ideology. J Sci Study Relig.

[30] Petersen LR, Donnenwerth GV (1997). Secularization and the influence of religion on beliefs about premarital sex. Soc Forces.

[31] Weller S, Davis K. Condom effectiveness in reducing heterosexual HIV transmission. Cochrane Database Syst Rev. 2002;(1):CD003255. doi:10.1002/14651858.CD003255. 10.1002/14651858.CD003255 PMC8407100 11869658

[32] Yotebieng M, Halpern CT, Mitchell EM, Adimora AA (2009). Correlates of condom use among sexually experienced secondary-school male students in Nairobi, Kenya. SAHARA J.

[33] Akwara PA, Madise NJ, Hinde A (2003). Perception of risk of HIV/AIDS and sexual behaviour in Kenya. J Biosoc Sci.

[34] Häggström-Nordin E, Hanson U, Tydén T (2002). Sex behavior among high school students in Sweden: improvement in contraceptive use over time. J Adolesc Health.

[35] Wang B, Hertog S, Meier A, Lou C, Gao E (2005). The potential of comprehensive sex education in China: findings from suburban Shanghai. Int Fam Plan Perspect.

[36] Markham CM, Tortolero SR, Peskin MF, Shegog R, Thiel M (2012). Sexual risk avoidance and sexual risk reduction interventions for middle school youth: a randomized controlled trial. J Adolesc Health.

[37] Bastien S, Kajula L, Muhwezi W (2011). A review of studies of parent–child communication about sexuality and HIV/AIDS in sub-Saharan Africa. Reprod Heal.

[38] Mengistu B, Birhane Y. HIV prevalence and associated factors among university students of Dire Dawa University, Easter Ethiopia 2009. 22nd Annual Conference of the Ethiopian Public Health Association. October 31–November. Addis Ababa: EPHA; 2011.

